# Supplementary material for: Association between oral microbiome and five types of respiratory infections: a two-sample Mendelian randomization study in east Asian population
Source: Front Microbiol. 2024 Apr 10;15:1392473. doi: 10.3389/fmicb.2024.1392473 (PMC11039966; doi:10.3389/fmicb.2024.1392473)
Supplement: Supplementary file 1 [file Data_Sheet_1.zip › Supplementary Materials/MR plots of saliva/Tonsillitis/s__Lancefieldella_sp000564995_mgs_2440/forest.pdf]

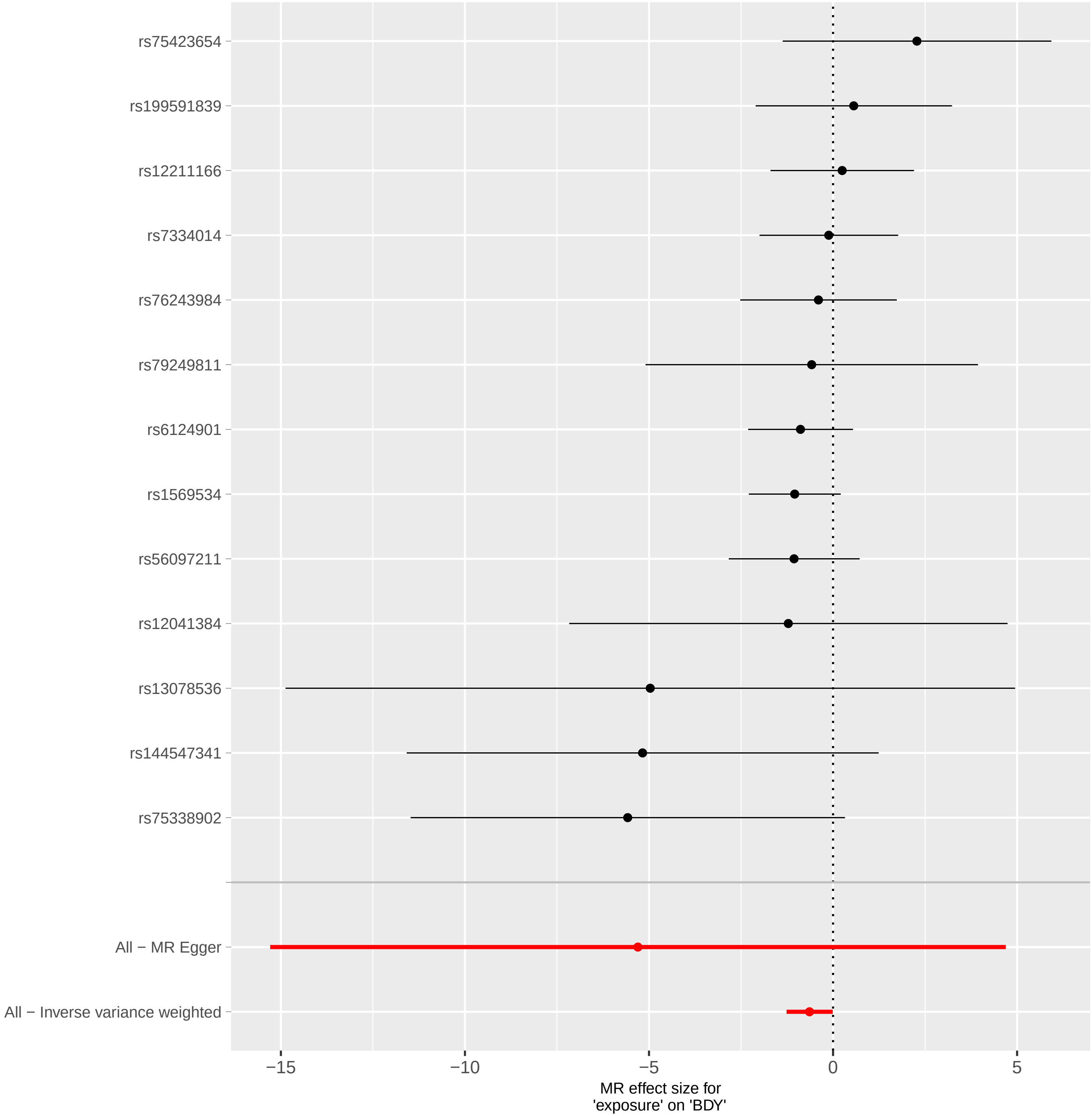

rs75423654
rs199591839
rs12211166
rs7334014
rs76243984
rs79249811
rs6124901
rs1569534
rs56097211
rs12041384
rs13078536
rs144547341
rs75338902
All − MR Egger
All − Inverse variance weighted
−15
−10
−5
0
5
MR effect size for
'exposure' on 'BDY'
